# Herpes simplex virus type-1 infection and spread in a novel porcine corneal explant model is restricted to the epithelium

**DOI:** 10.1371/journal.ppat.1013162

**Published:** 2025-05-02

**Authors:** Sana Arshad, Hafsa Rana, Naomi R. Truong, Ushasree Pattamatta, Kirstie M. Bertram, Andrew White, Holly R. Chinnery, Nicole A. Carnt, Anthony L. Cunningham

**Affiliations:** 1 Centre for Virus Research, The Westmead Institute for Medical Research, Westmead, New South Wales, Australia; 2 Centre for Vision Research, The Westmead Institute for Medical Research, Westmead, New South Wales, Australia; 3 Faculty of Medicine and Health, The University of Sydney, Sydney, New South Wales, Australia; 4 Department of Optometry and Vision Science, The University of Western Australia, Crawley, Western Australia, Australia; 5 School of Optometry and Vision Science, University of New South Wales, Kensington, New South Wales, Australia; 6 Institute of Ophthalmology, University College London, London, United Kingdom; University of Wisconsin-Madison, UNITED STATES OF AMERICA

## Abstract

Herpes Keratitis (HK) is a debilitating infection of the cornea that remains the leading cause of infectious blindness in developed countries. Caused primarily by herpes simplex virus type 1 (HSV-1), it is associated with recurrent inflammation, leading to corneal scarring. This study investigated the initial events during acute HSV-1 infection in the cornea by adapting our human anogenital mucosal explant model to a HSV-1 infected porcine corneal explant model. We infected these corneas topically via high-density microarray patches (HD-MAPs) dipped in GFP-labelled HSV-1. Virus infection and spread was detected by both GFP protein and RNAscope, adapted for HSV-1 DNA. The punctures were consistent, usually in the epithelium but some extended into the underlying stroma. However, HSV-1 was restricted to the corneal epithelium, without spread through the anterior limiting membrane (ALM) or Bowman’s layer into the stroma nor to the uppermost epithelial layer. This layer expressed SPRR1A similarly to the stratum granulosum of skin which is refractory to HSV-1 infection. In corneas where infected epithelial cells extended to the ALM, SPRR1A was also observed in this layer, suggesting it may contribute to its barrier function. Such studies of HSV-1 infection and spread will help improve therapy for HK and vaccine design to prevent it.

## Author summary

Herpes simplex keratitis is a leading cause of blindness and a major indication for corneal transplantation. To examine the earliest stages of infection and spread of herpes simplex virus type 1 (HSV-1) we developed a model system in pig corneas in explant culture, using a highly sensitive method for detecting HSV DNA, RNAscope and fluorescently labelled virus. Corneas from pigs more closely resemble those from humans in size and structure than mice. HSV-1 infection required intraepithelial delivery by microneedle patch punctures and the virus was shown to spread throughout the corneal epithelium except for the outermost layer. HSV-1 also did not spread beyond the anterior limiting membrane (ALM) into the subjacent stroma. SPPR1A, which contributes to the barrier function of cornified epithelium of skin, was expressed by the uppermost epithelial layer and, in infected cornea, observed in the ALM, suggesting it contributes to limiting both superficial and deep HSV-1 spread. The various successive stages of nuclear and cytoplasmic HSV-1 infection were shown in foci of infection by RNAscope. Techniques used in this model can be selectively applied in scarce human corneas to verify key findings and ultimately improve therapy for herpes keratitis and vaccine design to prevent it.

## Introduction

The cornea is a highly innervated, avascular, regularly arranged tissue designed to transmit and refract light for clear vision. It comprises three main layers: the epithelium, stroma and endothelium. The anterior limiting membrane (ALM) or Bowman’s layer is an acellular layer between the epithelium and stroma in the human and pig cornea that maintains its convex curvature [1]. Primary HSV-1 infection typically only involves the epithelium [2].

To examine the initial infection and spread of HSV-1 in detail, we adapted a recently developed foreskin explant model of HSV-1 infection used to map viral infection in human genital mucosa to porcine corneal explants [3]. There are morphological similarities between the upper layers of the skin/mucosa and the cornea. The most superficial layer of skin, the epidermis, is similar in cell composition and structure to the epithelium of the cornea, while the underlying layers of the dermis resemble the corneal stroma.

Novel features of this model are its use of high-density microarray patches (HD-MAPs) pre-treated with HSV-1 labelled on U_S_9 with green fluorescent protein (GFP), to simulate microtrauma during HSV infection, and the use of RNAscope *in situ* hybridisation to detect HSV DNA in infected cells and extracellular particles. RNAscope uses adjacently binding probes to specifically amplify target signals whilst also reducing background noise [4] and reveals the nuclear and/or cytoplasmic sites of infection at earlier stages than GFP expression and thus the precise boundaries of HSV spread. The HD-MAPs, originally intended for intradermal delivery of vaccines via microneedles [5], offer a consistent, reproducible method to infect the cornea compared to traditional methods including manual scarification with topical infection [6–8] or culturing in solution [9–13]. RNAscope and immunofluorescent (IF) microscopy for HSV-1-GFP were utilised together. The HD-MAPs created consistent punctures in the epithelium of the porcine cornea and successfully delivered HSV-1-GFP. Infection was localised to the epithelium only, despite the presence of a very fine ALM between epithelium and stroma [14] and evidence of some punctures penetrating deeper into the stroma. Furthermore, the uppermost epithelial layer was usually uninfected. Both this layer and the ALM expressed small proline-rich protein 1A (SPRR1A), a marker for the stratum granulosum in stratified squamous epithelia which is refractory to HSV-1 infection.

## Results

### Development of a novel corneal explant model to investigate the spread of acute HSV-1 infection

**Fig 1:**
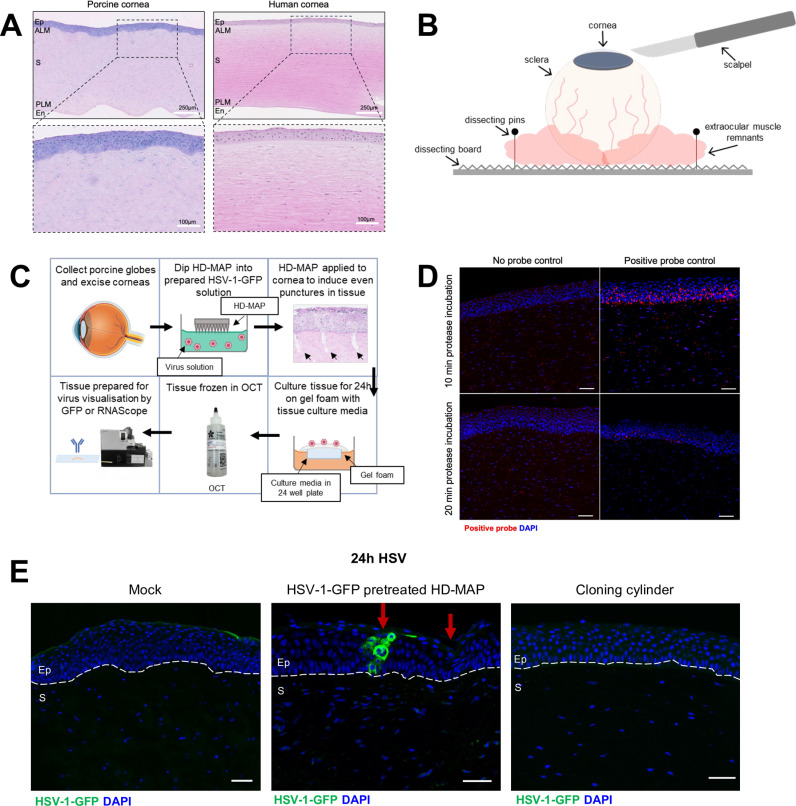
Development of a novel corneal explant model to investigate the spread of acute HSV-1 infection. **(A)** Histology of porcine and human cornea. Ep = epithelium, ALM = anterior limiting membrane, S = stroma, PLM = posterior limiting membrane, En = endothelium. **(B)** Set-up for the excision of the cornea from the porcine orbital globe. **(C)** Porcine corneas were subjected to HSV-1-treated (1 x 10^8^ PFU/mL) HD-MAPs or mock-treated HD-MAPs and cultured for 24 hours at 37˚C. Tissue was processed for RNAscope and IF staining. **(D)** Protease Plus digestion of 10 minutes for RNAscope was optimal in the porcine cornea for detection of positive control probe for Cyclophilin B RNA (red). **(E)** HSV-1-pre-treated HD-MAPs resulted in detectable infection of HSV-1-GFP, labelled using rabbit anti-GFP (1:1000) primary and donkey anti-rabbit AF488 (1:400) secondary (green) antibodies within the porcine cornea, whereas topical infection via cloning cylinders did not. Arrows = punctures. Scale bars = 50μm or as indicated. Schematic diagrams created in MS PowerPoint and BioRender.com.

A porcine corneal explant model was developed due to the similarities to human corneas (Fig 1A). Porcine corneas have an approximate central epithelial thickness of 80µm with six to eight cell layers compared to 50µm with five to seven cells layers in the human corneal epithelium [15–18]. Additionally, the porcine corneal endothelium is similar to the human corneal endothelium and comprises a single layer of hexagonal cells, vital for corneal clarity [14,17].

To characterise HSV-1 infection and spread in porcine cornea, an HSV-1 infection explant model previously optimised in anogenital tissue using HD-MAPs, pre-treated with HSV-1-GFP [3] was adapted to excised corneal explants (Fig 1B). HSV-1 was diluted in wetting agents to reduce surface tension, and applied with an automated applicator, to induce consistent microtrauma and mediate viral entry. Infection was determined by GFP labelling or RNAscope for HSV-1 DNA (Table 1, Fig 1C–1D).

**Table 1:** Summary of all porcine corneas infected with HSV-1, detected by GFP or RNAscope.

Animal	Cornea Sample	Mode of HSV-1 application	Treatment of animal before tissue harvest	HSV-1 detection by GFP	HSV-1 detection by RNAscope
1	3-L	HD-MAPs	Control - healthy	**+**	**–**
	3-R	Cloning cylinder		**–**	**–**
2	4-L	HD-MAPs	Platelet Derived Growth Factor-AB	**+++**	**+++**
	4-R				
3	5-L		Control - healthy	**+**	**+**
	5-R			**–**	**–**
4	6-L		Control - healthy	**+**	**+**
	6-R			**++**	**+++**

L = left, R = right.

- = no infection detected, + = low infection detected, ++ = mild infection detected, +++ = high infection detected.

Microtrauma was necessary for infection to occur. Delivery of HSV-1 to the porcine cornea via pre-treated HD-MAPs was successful (Fig 1E). The red arrows indicate punctures, including one over the GFP+ focus. In contrast, topical HSV-1 infection via cloning cylinders [19] glued to the corneal surface of the porcine explant showed no infection within epithelium or stroma (Fig 1E). Together, these results show that a novel corneal explant model using HD-MAPs can successfully and reproducibly induce acute infection in the porcine cornea.

### Detection of HSV-1 DNA via GFP and RNAscope in porcine corneal epithelium

**Fig 2:**
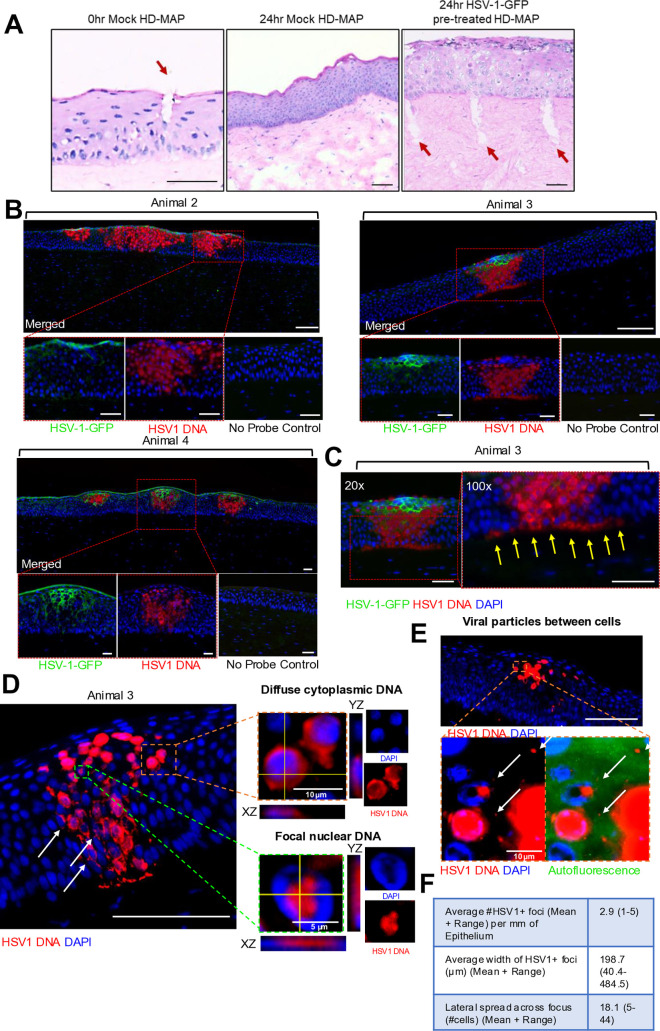
Detection of HSV-1 DNA via GFP and RNAscope in porcine corneal epithelium. Punctures in the patch-treated porcine corneal epithelium mostly healed by 24 hours but some penetrated into the stroma and were still obvious at this time. **(B)** Porcine corneas were subjected to HSV-1-treated (1 x 10^8^ PFU/mL) or mock treated HD-MAPs and cultured for 24 hours and stained for HSV-1-GFP (green) and HSV-1 DNA (red) via RNAscope as described earlier. Representative images of merged HSV-1-GFP and HSV-1 DNA shown in three animals. The superficial epithelial cells are only well defined in Animal 4, under autofluorescence marking of the upper boundary. In Animals 2 and 3 microfolding obscures the upper layer of epithelial cells with flattened nuclei **(C)** Representative image of 20x and 100x magnification of patch-treated porcine corneal epithelium. Yellow arrows indicate HSV-1 DNA spreading along basal epithelium of the cornea with no penetration into the stroma. Scale bar = 50μm. **(D)** High magnification (100x) images of various stages of infection as indicated by HSV-1 DNA expression, including insets with orthogonal views. Insets show 1) diffuse cytoplasmic and 2) focal nuclear HSV-1 DNA staining. Arrows show focal cytoplasmic infection. Scale bar = 100 µm or as indicated. **(E)** High magnification image of individual/aggregate HSV-1 particles (white arrows) which may be exiting or between infected cells. Autofluorescent channel indicates epithelium is intact. Scale bar = 100 µm or as indicated. **(F)** Table summarizing the average number of HSV-1 DNA+ foci per mm of epithelium, the average width of each foci (µm) and the lateral spread as number of cells across the focus, (mean + range).

Histological analysis of the porcine cornea immediately after patch application showed punctures in the epithelium which closed by 24 hours (Fig 2A). In some explants microneedle punctures penetrated beyond the ALM into the stroma. The optimised RNAscope protocol was then performed on infected porcine cornea to detect HSV-1 DNA. Using both IF and RNAscope allowed simultaneous observation of early and late stages of HSV-1 infection of epithelial cells. GFP was tagged to U_S_9, a protein that is expressed cytoplasmically at later stages of infection [20].

Porcine corneas were set up with HSV-1 or mock infection for 24 hours (Fig 2B). RNAscope showed much larger foci of infection extending beyond GFP staining, and various stages of cellular infection, focal or diffuse nuclear, with or without cytoplasmic staining (Fig 2D). HSV-1 was detectable within the corneal epithelium, and was absent from the subjacent stroma, despite the thin ALM and, in some samples, punctures extending into the stroma (Fig 2C). HSV-1 foci spread to 18.1 cells on average in 24 hours (Fig 2F). Where the upper epithelial surface was marked by autofluorescence (or, much less likely, leaked HSV-1-GFP), as in the Animal 4 inset, no infection of the most superficial layer of epithelial cells with flattened nuclei was clearly shown. Higher magnification imaging confirmed that there were no stromal cells infected with HSV-1 DNA nor free viral particles in the stroma, although free virus particles were observed either exiting from, or between epithelial cells (Fig 2E).

### SPRR1A is detected in the superficial epithelial layer of the porcine cornea

**Fig 3:**
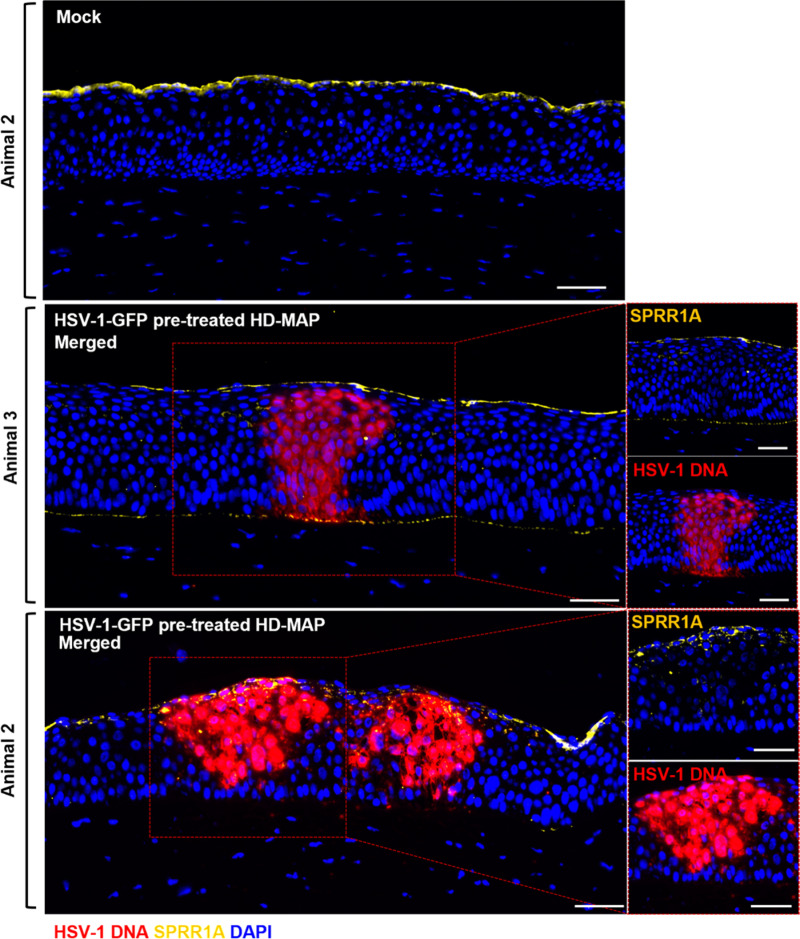
SPRR1A detected at the superficial and lower most epithelial layer of the porcine cornea. Porcine corneas were subjected to mock conditions or HSV-1-treated (1 x 10^8^ PFU/mL) HD-MAPs and cultured for 24 hours. Tissue sections were labelled for HSV-1 DNA via RNAscope (red), SPRR1A (1:40) primary and donkey anti-rabbit AF647 secondary (1:400) (yellow) antibodies. Representative image of merged HSV-1 DNA and SPRR1A in two animals shown. Scale bar = 50μm.

As HSV-1 infection was confined within the porcine corneal epithelium, and restricted in the superficial epithelial cell layer, we investigated the expression of SPRR1A, a protein associated with barrier function in skin keratinocytes where it is usually expressed in the stratum granulosum [21]. HSV-1 infected porcine corneas were stained to detect SPRR1A and HSV-1 DNA (Fig 3). Anti-SPRR1A stained the superficial epithelial cell layer, which had more flattened nuclei and was not infected by HSV-1. Unexpectedly, where HSV-1 infected cells in the foci extended to the ALM, SPRR1A staining was also seen in this layer adjacent to and extending laterally beyond the infected cells (Fig 3). In contrast, there was no SPRR1A staining of the ALM in mock samples or under HSV-1 DNA foci that did not extend to the ALM.

## Discussion

This study demonstrated initial HSV-1 infection and spread in porcine corneal explants as a novel model for acute human herpes simplex keratitis. Previous human corneal explant models have achieved successful epithelial HSV-1 infection [6,7,9,11–13]. However, only Courrier et al. showed evidence of HSV spread in intact epithelium using an active storage machine [7]. Their viral delivery system was not automated and their detection methods did not demonstrate viral infection in epithelial cell layers at various stages of infection. Porcine corneas are similar to the human cornea [22] and are more accessible compared to scarce donor tissue.

Here, delivery by HD-MAPs pre-treated with HSV-1-GFP, induced reproducible punctures in the porcine corneal epithelium which resulted in successful HSV-1 infection and spread limited to the epithelium, shown by both HSV-1-GFP and more sensitive RNAscope staining. HSV-1-GFP was also topically applied via cloning cylinders without microtrauma. No infection occurred indicating that microtrauma was required for viral entry in this model, although other HSV-1 strains need testing [10]. RNAscope showed much larger foci of infection extending beyond GFP staining, validated by no signal in negative controls. As in genital epithelium, RNAscope showed the early and successive stages of the virus life cycle in cells in infectious foci. These stages included focal nuclear staining, representing initial localisation of HSV-1 DNA to nuclear domains 10, more diffuse nuclear staining representing spread of nucleocapsids in the nucleus and cytoplasmic staining after exit from the nucleus [23,24]. GFP-tagged U_S_9 was translated later in the cytoplasm [3]. Thus, many cells were HSV-1 DNA-positive but GFP-negative, especially at the periphery of infected foci.

Like anogenital epithelium, RNAscope stained viral particles were occasionally observed either exiting or between epithelial cells [3], as in Courrier et al. who observed free enveloped virions above the ALM by electron microscopy, where epithelial cells were less dense [7]. This and the rapid growth of HSV-1 foci (18.1 cells wide) over 24 hours suggests both cell to cell and extracellular spread, as in genital mucosa. HSV-1 DNA-positive cells spread along the basal layer of the epithelium, but no free viral particles or infection of stromal cells was observed below the ALM.

Furthermore, the thin layer of the most superficial corneal epithelial cells with flattened nuclei were uninfected, analogous to infected human genital mucosal epithelial explants where epidermal infection and spread could only be achieved by penetration of HD-MAP punctures below the refractory stratum granulosum [3]. Primary HK is limited to the epithelium [2] and biopsies of recurrent HSV also show that infection is limited to the epidermis [25,26]. As foreskin fibroblasts and corneal keratocytes can be infected when grown *in vitro,* this suggests that the corneal ALM and skin/mucosal basement membranes usually limit deep spread [2].

The most superficial corneal epithelial cells also expressed SPRR1A. SPRR1A is one of several proteins found in the stratum granulosum of skin epidermis and orogenital mucosa [3,27] which are crosslinked to form the cornified cell envelope [28,29]. These are distributed similarly in human cornea [30], consistent with their function as a superficial barrier to HSV-1 infection. The finding of SPRR1A being contiguous with the ALM of infected but not normal cornea is intriguing and suggests local production by adjacent infected epithelial cells and concentration in the layer. Inflammation is known to induce SPRRs [28].

## Conclusions

HD-MAPs are a novel method of reproducibly infecting the corneal epithelium with HSV-1, suggesting microtrauma is required for initial corneal infection. RNAscope targeting HSV-1 DNA was more sensitive than GFP-labelled HSV-1, showing more extensive spread, larger foci and earlier and multiple stages of infection. The techniques optimised in this model can now be applied to efficiently study scarce human corneal explants, including initial interactions with resident immune cell subsets, using multidimensional techniques.

## Methods

### Ethics

All Study protocols were approved by the Western Sydney Local Health District Animal Ethics Committee.

### *Ex vivo* corneal infection model using pre-treated HSV-1-GFP HD-MAPs

Explanted porcine corneas were infected with HSV-1-U_S_9-GFP via HD-MAPs, (Vaxxas, Australia) or topically via a cloning cylinder glued to the corneal surface and then cultured, as described previously [3]. Explants were removed using forceps, frozen in OCT and sectioned with an HM505 cryostat (Microm Int. GmbH) in preparation for Haematoxylin & Eosin stain, RNAscope and IF microscopy described in S1 Data. All images were acquired as described previously [3].

### RNAscope and immunofluorescence staining of corneal tissue

Detection of HSV-1 DNA was conducted using the RNAscope 2.5HD Red Reagent Kit (ACD Bio) with probes specific for HSV1, as described [3]. Sections were then labelled with rabbit anti-GFP (polyclonal, Abcam, UK) and rabbit anti-SPRR1A (polyclonal, LSBio, USA) recognising porcine and human SPRR1A. Slides were stained with secondary antibodies as in figure legends. Slides were washed, nuclear stained, mounted, sealed and imaged as described in the previous protocol [3].

## Supporting information

S1 Data Document containing supplementary methods used to generate the experimental data in this paper. (DOCX)

S2 Data Excel spreadsheet of raw data used to generate Fig 2F. (XLSX)
