# Supplementary material for: Herpes simplex virus type-1 infection and spread in a novel porcine corneal explant model is restricted to the epithelium
Source: PLoS Pathog. 2025 May 2;21(5):e1013162. doi: 10.1371/journal.ppat.1013162 (PMC12068712; doi:10.1371/journal.ppat.1013162)
Supplement: S1 Data — (DOCX) [file ppat.1013162.s001.docx]

**Supplementary methods**

**Viruses**

HSV-1 stocks were prepared using Vero cells grown in 150 cm^2^ flasks in DMEM supplemented with 2% FBS. HSV-1 strain F expressing GFP-U_S_9 (HSV-1-GFP; donated by Prof. Renato Brandimarti (19) was propagated in Vero cells over two days. Cells were then harvested, and freeze-thawed three times. Sonication was performed using the Branson 450 Cup Digital Sonifier (400 W) three times for 20 seconds each with 70% amplitude and 40 second intervals between pulses to release the cell-associated virus. Cellular debris was pelleted by centrifuging at 4000 xg for 10 minutes at 4°C. Supernatants were stored at -80°C. Virus infectivity was determined via plaque assays using Vero cells where plaques were manually counted and the plaque-forming units (PFU) were calculated.

**RNAscope and immunofluorescence staining of porcine cornea**

Porcine eyes were collected after the harvest of organs after experiments from collaborators at the Westmead Institute for Medical Research (Table 1).

Detection of HSV-1 DNA was performed using the RNAscope 2.5HD Red Reagent Kit (ACD Bio) as per the RNAscope 2.5HD Manual protocol with a readily available probe against HSV-1 DNA targeting *UL30* (Cat no. 498861). Briefly, frozen tissue sections were fixed in 2% PFA for 20 minutes at room temperature (RT), treated with BLOXALL (Vector Laboratories, USA) for 10 minutes, followed by a 10-minute Protease Plus treatment (diluted 1:5 in cold PBS) and a 2-hour incubation with the HSV-1 DNA probe at 40°C performed in a HybEZ hybridisation oven (220VAC). No probe controls in which no target probes were added also confirmed the specificity of the probe signal. Slides were then subject to six rounds of amplification according to the manufacturer’s protocol, followed by a 1.5 minute development step with FastRed A+B substrate solution. Sections were labelled with primary antibodies overnight at 4°C in the dark. Slides were washed twice in 1xTris Buffered Saline (TBS) for 5 minutes total and subject to secondary antibody staining for 30 minutes at room temperature in the dark. Slides were washed as above, followed by 4’,6-diamidino-2-phenylindole (DAPI) nuclear stain at 1:1000 for 3 minutes, mounted with 20 x 24 mm rectangle coverslips (Menzel-Glašer, Germany) using SlowFade Diamond Antifade (Molecular Probes, USA), and sealed with nail polish. The images were acquired on the Olympus VS120 Virtual Slide Microscope and VS200 Slidescanner using 20x and 100x objectives.
